# Physiological and psychological symptom management based on electronic patient-reported outcomes: the TD-WELLBEING randomized clinical trial

**DOI:** 10.1038/s41416-025-03110-5

**Published:** 2025-08-07

**Authors:** Pengyu Jing, Ying Liang, Zhijun Tan, Xiaolong Yan, Jie Lei, Yunfeng Ni, Ximing Zhu, Chun Qiu, Jian Wang, Peng Ge, Yong Zhang, Lv Wang, Nan Zhao, Yong Zhang, Juan Wang, Yan Wang, Chunlong Zheng, Qiongjie Shao, Haiyue Zhang, Zhe Yang, Haichao Li, Jiangjiang Fan, Siming Liu, Kyriacos Kyriacou, Lei Shang, Zhongping Gu

**Affiliations:** 1 Department of Thoracic Surgery, The Second Affiliated Hospital of the Fourth Military Medical University, Xi’an, China; 2 Department of Health Statistics, Ministry of Education Key Lab of Hazard Assessment and Control in Special Operational Environment, the Fourth Military Medical University, Xi’an, China; 3 Department of Cerebral Surgery, The Second Affiliated Hospital of the Fourth Military Medical University, Xi’an, China; 4 Department of Thoracic Surgery, The Second Affiliated Hospital of Xi’an Medical College, Xi’an, China; 5 Department of Thoracic Surgery, The Affiliated Hospital of Shaanxi University of Traditional Chinese Medicine, XianYang, China; 6 Department of Thoracic Surgery, Xi’an Honghui Hospital, Xi’an, China; 7 Department of Thoracic Surgery, Daxing Hospital, Xi’an, China; 8 Department of Public Health, Xi’an Medical University, Xi’an, China; 9 Department of Pulmonary Medicine, Xijing Hospital, the Fourth Military Medical University, Xi’an, China; 10 Department of Economics Finance and Accounting, Brunel Business School, Brunel University of London, London, UK

**Keywords:** Lung cancer, Surgical oncology, Lung cancer

## Abstract

**Background:**

One-third of all lung cancer cases globally are reported in China. This study evaluated the symptom management efficacy of an electronic patient-reported outcomes (ePRO)-based intervention for postoperative symptoms like pain and psychological distress after lung cancer surgery.

**Methods:**

We included lung cancer surgery patients (April 2022–October 2023; age, 18–75 years) with ECOG scores of 0–2 and expected survival of >6 months and randomized them into control and intervention groups. The latter completed MDASI-LC and QLQ-C30 questionnaires, wherein high symptom scores prompted treatment recommendations; the former received routine care. Changes in symptom scores, daily function, and quality of life were evaluated over 12 weeks and 1 year through surveys and interviews for ePRO-based symptom management efficacy assessments.

**Results:**

Herein, 355 participants comprised intervention (*n* = 182) and control groups (*n* = 173). At 12 weeks, the former had significantly lower symptoms threshold [0 (0–1) *vs*. 1 (0–3)], lower symptom scores [adjusted mean difference, −0.527 (95% CI: −0.788 to −0.266)], and higher QOL scores (emotional function: 2.908; 95% CI: 0.600–5.216, *P* = 0.014; global health: 6.775; 95% CI: 3.967–9.583).

**Conclusions:**

ePRO-based collaborative management effectively lessened postoperative burden and improved QOL beyond 6 months.

## Introduction

Lung cancer, a leading cause of cancer-related deaths, leaves many patients with a significant symptom burden postoperatively [1, 2], affecting their quality of life (QOL) [3, 4]. Although hospital stays are brief (typically 6–8 days), effective symptom management after discharge is critical yet underemphasized in patient care.

WeChat (Tencent, Shenzhen, China) is a multi-purpose platform integrating instant messaging, social networking, mobile payments, and mini-programs; it offers a promising solution for ongoing post-discharge symptom management [3, 5], enabling healthcare providers to communicate continuously with patients, offer timely support, monitor symptoms, and deliver personalized interventions. This enhanced engagement can improve both the physiological and psychological recovery of patients, reducing distress and enhancing overall QOL.

Symptom management is a cornerstone of healthcare [6]. Electronic patient-reported outcomes (ePRO), which capture health information directly from patients via electronic means, reportedly improve patient satisfaction [7, 8]. and clinical outcomes [7, 9]. Although ePRO use is growing in surgical settings, few studies address both physiological and psychological symptoms [2, 3, 10]. Its growing use aims to assess sadness and depression, addressing which is vital to improve QOL and survival rates [11]. Comprehensive ePRO-based postoperative care could bridge this gap by integrating psychological symptom management postoperatively.

This study evaluates an ePRO-based symptom management model tailored to the specific physiological and psychological needs of lung cancer patients postoperatively. The interventions include (1) digital platforms for remote monitoring, symptom reporting, and interaction; (2) multimodal physiological symptom management; (3) cognitive-behavioral therapy for psychological symptoms; and (4) supportive care like nutrition, exercise, and social support. With ePRO, healthcare providers gain real-time access to patients’ experiences, enabling personalized care and prompt medical decision adjustments, which can reduce symptom burden, improve QOL, and optimize resources.

Using a WeChat-based ePRO model, we tracked symptoms and QOL in lung cancer patients at 12 weeks and one year postoperatively, and compared outcomes with those receiving routine care to assess whether the ePRO model demonstrated a more beneficial impact on holistic recovery.

## Methods

### Trial design and participants

This RCT was conducted across four tertiary hospitals in China [12] and registered with the Chinese Clinical Trial Registry (ChiCTR2200058876). All procedures received ethical approval, and informed consent was obtained from each participant, ensuring study compliance with the Declaration of Helsinki. All methods were performed in accordance with the relevant guidelines and regulations. Participants were 18–75 years old; they had high suspicion of lung cancer on imaging, an Eastern Cooperative Oncology Group score of 0–2, and an estimated survival period of >6 months. Exclusion criteria were receiving neoadjuvant therapy, a history of psychotropic drug use or abuse, and inability to understand the study.

### Randomization and blinding

Independent statisticians used SAS 9.4 PROC PLAN to generate random numbers for participant grouping. The randomization table was integrated into the WeChat applet, assigning patients to the control or intervention group (1:1). A third-party statistician provided the randomization scheme, and the clinical staff assigned study numbers’ eligibility, linking each participant to a specific group. To minimize measurement bias, all data collectors and analysts were blinded to group assignments.

### Intervention

Participants completed the MD Anderson Symptom Inventory-Lung Cancer module (MDASI-LC) questionnaire via a WeChat mini program at these intervals: before the operation, postoperative days 1, 3, 5, and 7, and then weekly until 12 weeks postoperatively. They also completed the EORTC QLQ Core Questionnaire (QLQ-C30) on paper before the operation and at 4 and 12 weeks postoperatively. For target symptoms scoring ≥4 (e.g., cough, pain, sleep issues, fatigue, shortness of breath, depression, and sadness), the intervention group received standardized treatment advice via WeChat [12]. At the same time, the participating specialists (thoracic surgeons) received early notification messages on their mobile devices, prompting them to contact the patient via WeChat or phone within 24 h. They offered management advice based on the ePRO score, which may have included recommendations about whether a consultation is needed, educational resources, medication guidance, and assessing the necessity for the patient to visit the emergency room, clinic, or hospital. In addition, if the patients reported any other symptoms or if their ePRO score exceeded the established threshold, standard treatment recommendations created by lung cancer specialists appeared on the WeChat ePRO interactive interface. Doctors provided management guidance within 24 h, when two or more symptoms had scores >6. Psychiatrists monitored psychological symptoms, offering recommendations for scores 4–6 and telephonic consultations for scores >6. In this study, standardized management guidance was developed by five senior lung cancer experts on the basis of relevant standards, such as the Chinese Lung Cancer Diagnosis and Treatment Standards (2021 Edition), ECOG-ACRIN Lung Cancer Treatment Guidelines, postoperative pain management standards, postoperative cough management, and the combination of postoperative activities and functional exercises, combined with clinical experience. Two clinical psychologists with 10 years of total clinical experience were invited to provide standardized response suggestions for psychological symptoms. In addition, standardized treatment standard training was provided to doctors and nurses participating in the trial. After standardized training, they were proficient in postoperative management of lung cancer patients. To ensure that clinicians responded to symptom alerts within 24 h, daily alarms were set at two fixed time points (17:30 and 20:30) on their phones, reminding them to process patient alerts promptly. This mechanism ensured timely handling of symptom notifications.

### Routine care

Control group patients received routine management without WeChat symptom alerts, the data collection time and method were the same as those of the intervention group. Each research center provided symptom counseling services, and patients decided if they needed medical assistance based on their symptoms. If needed, they could access long-term free medical counseling via WeChat to support their health. Patients in the routine care group could contact doctors and staff at any time through WeChat groups, which is more convenient for obtaining medical knowledge and assistance compared to going to the hospital. Both groups of patients would receive a discharge guide upon discharge, which includes instructions for protecting the surgical incision and removing stitches, suggestions for postoperative lifestyle, treatment and follow-up, and guidance on the return time of paper questionnaires.

### Outcome measures

The primary outcome was the average number of target symptom threshold events at 12 weeks postoperatively (on the day of filling out the form), covering cough, pain, sleep disorders, fatigue, sadness, shortness of breath, and depression. A score of 4–6 was deemed moderate and >6 was severe, without a threshold score of <4 triggering intervention.

Secondary outcomes included ePRO trajectory changes in symptom score, daily function, and QOL. PROs were defined as (1) the average score for the seven targeted symptoms, (2) the average score of six MDASI-LC daily functional items, and (3) QOL from the EORTC QLQ-C30 questionnaire. Functional scores covered physical (general activity, work, and walking) and affective interference (mood, relations, and enjoyment of life). MDASI-LC was tracked from baseline to 12 weeks postoperatively (before and 1, 3, 5, and 7 days after operation and weekly until 12 weeks postoperatively), QLQ-C30 questionnaire was investigated only baseline, 4 and 12 weeks postoperatively.

Subgroup analysis were be performed on separate samples of patients receiving (not receiving) adjuvant therapy.

Exploratory objectives assessed doctor’s workload, patient acceptance, and satisfaction. Patient satisfaction was surveyed, focusing on WeChat convenience and expert guidance. In addition, long-term changes in symptom scores, daily function, QOL, and threshold events were evaluated at 6 months and 1 year, with the trajectory drawn from the average of the seven symptoms and MDASI-LC items for daily function and the QOL derived from QLQ-C30.

### Statistical analysis

Sample size was calculated using an independent sample Student’s t-test (α = 0.05, 1−*β* = 0.8). To detect a minimum important difference (0.3*SD) in average symptom threshold events, 176 participants per group were needed. Assuming a 20% dropout rate, the minimum sample size was 220 per group.

The intention-to-treat (ITT) analysis set included all the randomized subjects, and the per protocol set (PPS) included all the subjects followed the protocol, excluding those failed to comply with intervention measures twice, experienced serious complications, could not complete the ePRO questionnaire, had poor compliance, or requested withdrawal.

The analysis mainly included ePRO data at 16 time points: before the operation, postoperative days 1, 3, 5, and 7, and then weekly from 2 to 12 weeks. In addition, two additional time points, 6 months and 12 months after operation, were added. Due to non-normal data distribution, the Wilcoxon–Mann–Whitney test was used for primary comparisons, and a linear mixed-effects model analyzed ePRO score changes over time for secondary outcomes, with patients and time as random effects. Additional analyses used Fisher’s exact test, Chi-square test, or descriptive statistics.

The main analysis was based on the PPS set, with significance set at a bilateral *P* value of ≤0.05. Analyses were conducted in SAS 9.4, and all figures were created using R 4.4.1.

## Results

**Fig. 1:**
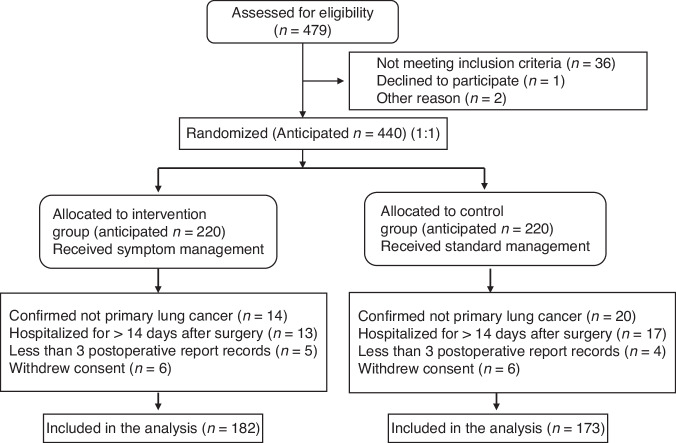
CONSORT diagram.

Of the 479 patients evaluated from April 2022 to October 2023, 36 did not meet the inclusion criteria, 1 refused participation, and 2 withdrew for other reasons. A total of 440 patients were randomly assigned to intervention and control groups (1:1, 220 per group). After randomization, 38 intervention and 47 control group patients met the withdrawal criteria. The final analysis included 182 and 173 patients in intervention and control groups, respectively (Fig. 1).

### Baseline characteristics

Table 1 presents the clinical and demographic characteristics of the patients, showing no significant differences between the two groups. The average age was similar (control: 56.83 ± 9.64 years *vs*. intervention: 55.60 ± 10.52 years), and the median hospital stay was 7 days in both groups.

**Table 1:** Baseline characteristics.

Participant characteristics	Control group (*n* = 173)	Intervention group (*n* = 182)	*P*-value
Age (years)			0.542
<60	99 (57.23)	110 (60.44)	
≥60	74 (42.77)	72 (39.56)	
Sex			0.867
Male	107 (61.85)	111 (60.99)	
Female	66 (38.15)	71 (39.01)	
Family income (yuan)			0.742
≥10,000	18 (10.40)	17 (9.34)	
< 10,000	155 (89.60)	165 (90.66)	
Educational level			0.987
≤High school	118 (68.21)	124 (68.13)	
>High school	55 (31.79)	58 (31.87)	
Marital status			0.512
Single (Divorced/Widowed)	26 (15.03)	23 (12.64)	
Married	147 (84.97)	159 (87.36)	
Smoking status			0.988
Never	116 (67.05)	122 (67.03)	
Current or former	57 (32.95)	60 (32.97)	
Insurance			0.723
Employee Medical Insurance	120 (69.36)	121 (66.48)	
Resident Medical Insurance	48 (27.75)	57 (31.32)	
Commercial Insurance	5 (2.89)	4 (2.20)	
Patient source			0.823
Other hospitals	5 (2.89)	6 (3.30)	
Tangdu hospital	168 (97.11)	176 (96.70)	
Family history of lung cancer			0.281
No	145 (83.82)	160 (87.91)	
Yes	28 (16.18)	22 (12.09)	
Comorbidity (CCI)			0.557
0	11 (6.36)	16 (8.79)	
1–2	71 (41.04)	81 (44.51)	
3–4	77 (44.51)	75 (41.21)	
5–6	14 (8.09)	10 (5.49)	
Extent of surgery			0.524
Sublobar	27 (15.61)	33 (18.13)	
Lobe	146 (84.39)	149 (81.87)	
Histology			0.537
Non-adenocarcinoma	13 (7.51)	16 (8.79)	
Adenocarcinoma	160 (92.49)	166 (91.21)	
Stage			0.844
I	157 (90.75)	165 (90.66)	
II-III	16 (9.25)	17 (9.34)	
Postoperative adjuvant therapy			0.595
No	155 (89.60)	167 (91.76)	
Yes	18 (10.40)	15 (8.24)	
ECOG			0.953
0	171 (98.84)	180 (98.90)	
1–2	2 (1.16)	2 (1.10)	
Surgical approach			0.365
Thoracoscopy	169 (97.69)	180 (98.90)	
Thoracotomize	4 (2.31)	2 (1.10)	
Hospital time (day) [M (*P*_25_, *P*_75_)]	7 (6, 9)	7 (6, 10)	0.391

Except for age and hospital time, the rest are presented as n (%).

*CCI* Charlson Comorbidity Index, *ECOG* Eastern Cooperative Oncology Group.

### Symptom threshold events

At each postoperatively time point, the number of subjects included in the PPS analysis at each time point is shown in the supplementary material (Supplementary Material, Table S1.1 and Table S1.2). During this period, the intervention group recorded 4762 symptom threshold events and 1469 alerts, with each alert representing 1–7 symptom threshold events. There were 718 emotional symptom threshold events (depression or sadness >4) and 178 psychological consultations by psychologists (depression or sadness >6). In addition, 250 interventions by thoracic doctors (two or more symptoms >6 exclude depression and sadness).

### Primary and secondary outcomes

**Fig. 2:**
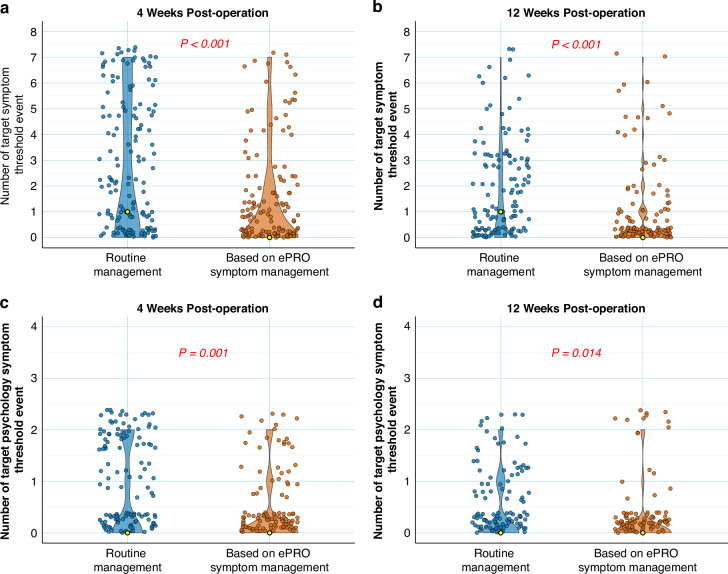
Comparison of postoperative target symptom threshold event at 4 weeks and 12 weeks. (**a**, **b**) and comparison of severe psychological threshold event at 4 weeks and 12 weeks (**c**, **d**). The violin plot displays the median (yellow dot in the box), 25th and 75th quartiles (box limits), and scatter point representing the data distribution.

For the seven target symptoms, the intervention group had significantly fewer symptom threshold events than the control group at both 4 weeks [0 (0–2) *vs*. 1 (0–5), *P* < 0.001] (Fig. 2a) and 12 weeks [0 (0–1) *vs*. 1 (0–3), *P* < 0.001] (Fig. 2b) postoperatively. At 4 weeks, the intervention group also showed fewer threshold events for psychological symptoms (sadness or depression, score >4) compared to the control group [0 (0–0) *vs*. 0 (0–2), *P* = 0.001] (Fig. 2c). At 12 weeks, the difference of threshold events between the two groups was still significant [0 (0-0) *vs*. 0 (0-1), *P* = *0.014*] (Fig. 2d).

**Fig. 3:**
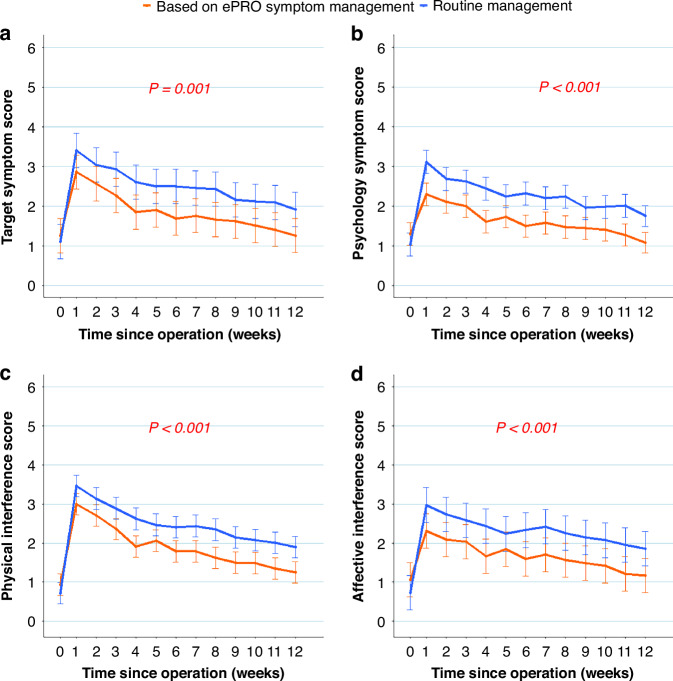
Symptom severity over time. **a** Postoperative symptom score of the target symptoms (pain, fatigue, disturbed sleep, shortness of breath, coughing, sadness and depression). **b** Postoperative psychological symptom score (sadness and depression). Higher scores indicate more severe symptoms. **c** Physical interference score (MDASI-LC: general activity, work, and walking). **d** Affective interference score (MDASI-LC: mood, relations with others, and enjoyment of life). Higher scores indicate more greater functional interference. MDASI-LC MD Anderson Symptom Inventory-Lung Cancer module. Error bar represents 95% CI of adjusted mean difference. ePRO electronic patient-reported outcome. 0 represents baseline (within 1 week before surgery).

At 12 weeks postoperatively, the intervention group had significantly lower symptom scores than the control group for the seven target symptoms (adjusted mean difference, −0.527; 95% CI: −0.788 to −0.266; *P* < 0.001; Fig. 3a) and for sadness and depression (-0.601; 95% CI: −0.909 to −0.293; *P* < 0.001; Fig. 3b). The former had significantly lower physical and affective interference scores as well [−0.528; 95% CI: −0.828 to −0.228; *P* = 0.006 (Fig. 3c) and −0.582; 95% CI: −0.854 to −0.281; *P* < 0.001 (Fig. 3d), respectively].

**Fig. 4:**
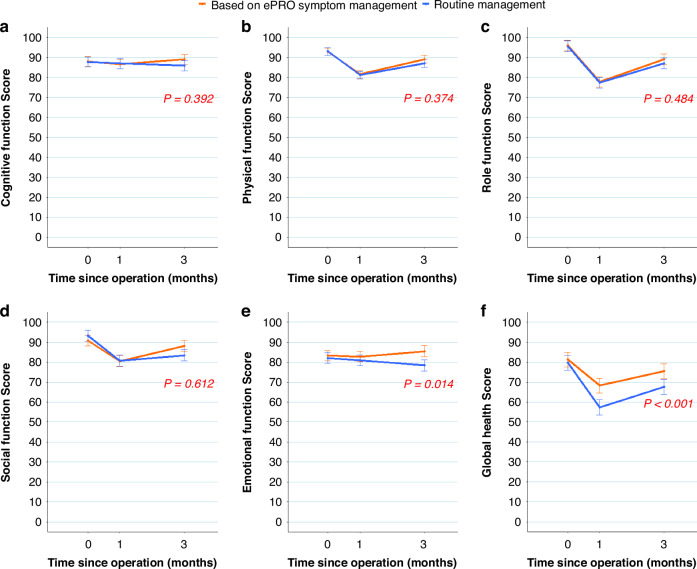
Quality of life (QLQ-C30) at 12 weeks postoperatively. **a** Cognitive function score. **b** Physical function score. **c** Role function score. **d** Social function score. **e** Emotional function score. **f** Global health score (average of overall health, where higher scores indicate better quality of life.). Error bar represents 95% CI of adjusted mean difference. QLQ-C30 EORTC core quality of life questionnaire; ePRO electronic patient-reported outcome. The Y-axis displays the standardized QLQ-C30 dimension score. 0 represents baseline (within one week before surgery).

At 12 weeks postoperatively, the intervention group showed significant improvements in emotional function and global health dimensions of QOL compared to the control group, whereas the differences in physical function (*P* = 0.36), cognitive function (*P* = 0.37), social function (*P* = 0.60), and role function (*P* = 0.47) were insignificant (Fig. 4a-d). Specifically, with the intervention, emotional function improved by 2.908 (95% CI: 0.600–5.216; *P* = 0.014; Fig. 4e) and global health improved by 6.775 (95% CI: 3.967–9.583; *P* < 0.001; Fig. 4f). Intention-to-treat analyses generated similar results as the per-protocol analyses for primary and secondary outcomes (Supplementary Material, Fig. S1 and Table S2).

### Other outcomes and feasibility report

At 12 weeks postoperatively, the subgroup analysis results were as follows: In patients who received adjuvant therapy, the intervention group had significantly fewer symptom threshold events than the control group [0 (0-0) *vs*. 3 (1-4), *P* = 0.003], with significant difference in psychological symptom threshold events ([0 (0-0) *vs*. 0.5 (0-1), *P* = 0.028]. Among patients not receiving adjuvant therapy, there was a significant difference in main symptom thresholds between the intervention and control groups [0 (0–1) *vs*. 0 (0–2), *P* = 0.001], with no significant difference also observed in psychological symptom thresholds events (*P* = 0.07).

The exploratory analysis results were as follows: At six months postoperatively, the intervention group had significantly fewer symptom threshold events for the seven target symptoms compared to the control group [0 (0-0) *vs*. 0 (0–1), *P* < 0.001]. However, by one year postoperatively, there was no significant difference in symptom threshold between the groups (*P* = 0.15). Throughout the year, symptom scores for sadness and depression remained significantly lower in the intervention group [−0.499; 95% CI: −0.737 to −0.260; *P* < 0.001 (Supplementary Material, Fig. S2A) and −0.451; 95% CI: −0.706 to −0.197; *P* < 0.001 (Supplementary Material, Fig. S2B)]. Physical and affective interference scores were also significantly lower in the intervention group over the year [−0.322; 95% CI: −0.555 to −0.089; *P* = 0.007 (Supplementary Material, Fig. S2C) and −0.345; 95% CI: −0.575 to −0.115; *P* = 0.003 (Supplementary Material, Fig. S2D)].

At one year postoperatively, the intervention group showed a significant improvement in emotional function (3.509; 95% CI: 0.709–6.309; *P* = 0.014; Supplementary Material, Fig. S3A) and global health (4.933; 95% CI: 2.718–7.148; *P* < 0.001; Supplementary Material, Fig. S3B), consistent with the 12-week results.

Doctors spend an average of 2 minutes handling each symptom alarm. The number of phone consultations rate is 17.01% (250/1469) for symptom alarm, psychologists’ telephonic consultations averaged 5 min (range: 3–18 min), and consultations rate is 24.79% (178/718). Patient satisfaction and acceptance of ePRO-based symptom management and WeChat Mini Program Interactive Platform were high (Supplementary Material, Table S3). The patient satisfaction survey had a response rate of 78.57% (143/182). All patients in the intervention group were satisfied with the mini program’s symptom management methods, with a median satisfaction score of 5 (range: 1–5). Most patients 87.41% (125/143) reported that completing the ePRO questionnaire caused little to no inconvenience in their daily lives (Supplementary Material, Table S4). For telephonic interventions by psychologists, 51.72% (74/143) of intervention group patients received calls, among them, 72.97% (54/74) felt completely relaxed after the call, 24.32% (18/74) felt somewhat relaxed, and 2.70% (2/74) reported no mood change (Supplementary Material, Table S4). The readmission rates of the intervention group and the control group were 1.64% and 5.78%, respectively, with the control group being higher than the intervention group (*P* = 0.038).

## Discussion

Dynamic monitoring and timely interventions for postoperative symptoms are crucial for early recovery and improved QOL in lung cancer patients [4, 5, 13]. Inadequate communication and delayed feedback on surgery-related issues can impede both physiological and psychological recovery. ePRO-based symptom management allows patients to report discomfort promptly, enhancing doctor-patient communication. Although ePRO-based management positively impacts physical symptoms in lung cancer patients postoperatively [1, 3, 5], its effectiveness and feasibility in managing psychological symptom remains unknown. Our findings indicate that ePRO-based management provides both physiological and psychological relief and enhances QOL in postoperative lung cancer patients. Furthermore, this care model showed high adoption rates among surgeons and psychologists and increased patient satisfaction in our study.

Unlike previous RCTs solely focused on physiological symptom monitoring in surgical settings [3, 6], we expanded ePRO use to include both psychological and physiological symptom management postoperatively, aiming for a more holistic approach. This study also fostered interdisciplinary collaboration between surgeons and psychologists. In addition, this RCT also evaluated the utility of ePRO over short-term (4 weeks), medium-term (12 weeks and 6 months), and long-term periods (1 year) periods following lung cancer surgery, providing a comprehensive perspective on ePRO-based symptom management. Our findings corroborate two prior studies [2, 3, 5], and this study is the first showing that patients receiving ePRO-guided intervention for both psychological and physiological symptoms experienced significantly improved QOL. Furthermore, 97.27% of postoperative lung cancer patients reported feeling relaxed following ePRO-based psychological interventions, with significantly reduced psychological symptom scores at 12 weeks postoperatively (*P* < 0.001), and these benefits extended up to 1 year.

The clinical benefits of the ePRO management model for lung cancer patients can be summarized as follows. First, as an early warning system, ePROs enable clinical intervention before the condition worsens, thus enhancing patient safety and optimizing medical resource usage [7, 11]. Second, ePROs improve patient satisfaction by allowing patients to track changes in their condition and treatment effectiveness, fostering their greater participation, proactivity, and overall satisfaction [14]. Third, ePRO-based remote care expands access, reduces costs, saves time, and lessens the resource demands associated with frequent in-person visits [15]. Finally, ePRO-based symptom management promotes interdisciplinary collaboration, facilitating tailored, holistic interventions and follow-up care [16].

From a psychological perspective, incorporating mental well-being in this RCT represents a notable advancement over prior studies, which explored ePRO-based management of only physiological symptoms [2, 3, 10]. Notably, compared to the control group, the intervention group had consistently lower psychological symptom scores and emotional function interference at all time points. It also had a lower symptom burden and improved overall health, surpassing previous findings [2, 3, 5]. However, these findings further underscore the pivotal role of incorporating psychological factors in modulating postoperative symptom burden and enhancing QOL. A striking 97.27% of patients reported emotional relief following psychological intervention, underscoring the vital role of psychologists in ePRO-based postoperative care for lung cancer, fostering patient engagement and satisfaction by mitigating emotional distress. Thus, integrating psychological monitoring and intervention is crucial, marking a novel advancement in postoperative care. Our study also involved interdisciplinary collaboration between thoracic surgeons and psychologists: surgeons managed surgical and physiological symptoms, while psychologists resolved mental health issues and provided emotional support. In the first 12 weeks postoperatively, 4762 symptom thresholds and 1469 alerts were generated in the intervention group. Surgeons responded to 250/1469 alerts, and psychologists intervened in 178/718 psychological alerts. To our knowledge, this is the first study in China to establish that such collaboration can relieve postoperative symptoms burden in lung cancer patients, promptly addressing psychological issues (e.g., sadness and depression) and improving QOL.

Regarding the postoperative support duration, this RCT had the largest sample size of data among related studies, covering short- (4 weeks), medium- (12 weeks to 6 months), and long-term (1 year) periods postoperatively. This scope enhances the reliability and generalizability of the results. Our key findings show that in the short- and medium-term, the intervention group had significantly lower symptom thresholds, symptom burden, physical interference, and emotional interference scores than the control group. Although no significant difference in symptom thresholds was observed at one year postoperatively, the intervention group maintained lower interference scores, suggesting the need for effective ePRO-based interventions with lasting benefits for postoperative recovery. However, the QLQ-C30 scale showed no significant differences between groups in social, cognitive, role, or physiological functional dimensions. This may be due to response shift—changes in patients’ internal cognitive appraisal caused by disease and surgery—which could have reduced the intervention’s apparent effectiveness [17, 18].

Although this ePRO model has impressive feasibility, it should not increase the workload of doctors [19, 20] but still maintain high patient satisfaction [21]. This ePRO-based approach automated standardized feedback and enabled swift physician response to alerts. Doctors embraced the model with a 100% acceptance rate and an average intervention time of 2 min for one patient; psychologists provided telephonic interventions with a response average call duration of 5 min. Notably, this model did not burden physicians and was well received by both surgeons and psychologists, highlighting its feasibility. Automated feedback and the doctor–patient interaction through the WeChat Mini Program were key to enhancing patient convenience [22, 23]. When symptom thresholds were exceeded, patients received tailored interventions, and doctors received alerts for immediate assessment and guidance via the WeChat ePRO system. This efficient interaction minimized intervention delays, reduced rehospitalization rate, conserved resources, and secured high patient satisfaction.

### Limitations

This study has some limitations: (1) The sample lacks comprehensiveness, focusing mostly on stage I patients with few stage II and III cases (16 and 18 cases, respectively), limiting stage-specific analyses and calling for broader samples across various stages. (2) The 1–12 months follow-up period may not fully capture long-term QOL effects, indicating a need for extended follow-ups at specific time points. (3) In this study, filling out the WeChat mini program questionnaire required a smartphone. In China, although most patients knew how to use a smartphone or could get assistance from a family member, a few patients still did not have or did do not know how to use a smartphone. Therefore, the conclusion is not applicable to this group of people. (4) Although thoracic surgeons and psychologists collaborated effectively, the former managed multiple aspects (e.g., pain, cough, rehabilitation, and tumor follow-up) that should typically be overseen by pain specialists, rehabilitation specialists, or oncologists; thus, future studies should assemble a multidisciplinary team for more specialized support. (5) Potential biases from unaccounted confounders and occasional patient exclusions highlight the need for rigorous controls [24], though the consistency between intention-to-treat and per-protocol analyses supports the study’s reliability. (6) Given the impact of the recent COVID-19 pandemic, the number of subjects from other hospitals except for Tangdu Hospital is small, and the results may thus have some bias. It is necessary to further study the situation of each hospital to verify the applicability of this ePRO-based symptom management model.

## Conclusions

This RCT showed that ePRO-based symptom management effectively alleviates postoperative physiological burdens and enhances mental well-being and QOL of lung cancer patients, with benefits evident in short-, medium-, and long-term periods. This model showed feasibility and efficacy in postoperative care, with high acceptance from surgeons, psychologists, and patients, establishing it as a recommended approach for comprehensive management both physiological and psychological symptoms in lung cancer patients.

## Supplementary information

Supplementary Material

## Data Availability

The datasets supporting this study are available from the corresponding authors upon reasonable request, in compliance with FAIR data principles (Findable, Accessible, Interoperable, Reusable). All data sharing activities adhere to relevant ethical guidelines and institutional review board approvals.
