# Supplementary material for: Physiological and psychological symptom management based on electronic patient-reported outcomes: the TD-WELLBEING randomized clinical trial
Source: Br J Cancer. 2025 Aug 7;133(7):937–44. doi: 10.1038/s41416-025-03110-5 (PMC12480890; doi:10.1038/s41416-025-03110-5)
Supplement: Supplementary file 1 — Supplementary Material [file 41416_2025_3110_MOESM1_ESM.doc]

**Table S1.1** The number of MDASI-LC cases included in the PPS

| **Time Points** | **Control** | **Intervention** |
| --- | --- | --- |
| Baseline | 173 | 182 |
| 1(1days) | 173 | 182 |
| 2(3days) | 171 | 180 |
| 3(5days) | 172 | 181 |
| 4(7days) | 170 | 181 |
| 5(2weeks) | 172 | 182 |
| 6(3weeks) | 172 | 181 |
| 7(4weeks) | 173 | 182 |
| 8(5weeks) | 173 | 180 |
| 9(6weeks) | 173 | 181 |
| 10(7weeks) | 173 | 180 |
| 11(8weeks) | 170 | 180 |
| 12(9weeks) | 172 | 180 |
| 13(10weeks) | 172 | 180 |
| 14(11weeks) | 173 | 180 |
| 15(12weeks) | 173 | 182 |
| 16(6months) | 172 | 182 |
| 17(12months) | 172 | 178 |

Abbreviations: MDASI-LC, MD Anderson Symptom Inventory-Lung Cancer Module

**Table S1.2** The number of QLQ-C30 cases included in the PPS

| **Time Points** | **Control-Available** | **Intervention-Available** |
| --- | --- | --- |
| Baseline | 173 | 182 |
| 7(4weeks) | 168 | 176 |
| 15(12weeks) | 165 | 168 |
| 16(6months) | 164 | 165 |
| 17(12months) | 164 | 164 |

Abbreviations: QLQ-C30, Research and Treatment of Cancer Quality of Life Questionnaire - Core 30

**Table S2** Intention-To-Treat Analyses for Secondary Outcomes of Symptom and Functional Scores over Time

Intervention Group *vs*. Control Group

|  | Estimate | 95% CI | | *P* |
| --- | --- | --- | --- | --- |
|  |  | lower | Upper |  |
| **MDASI-LC** |  |  |  |  |
| Target symptom score | -0.443 | -0.672 | -0.214 | 0.001 |
| Psychology symptom score | -0.397 | -0.689 | -0.105 | 0.008 |
| Physical interference score | -0.388 | -0.667 | -0.109 | 0.006 |
| Affective interference score | -0.422 | -0.685 | -0.160 | 0.002 |
| **QLQ-C30** |  |  |  |  |
| Emotional function | 3.865 | 1.699 | 6.031 | <0.001 |
| Global health | 6.775 | 4.205 | 9.346 | <0.001 |

NOTE. Target symptom score means average score of the seven target symptoms of pain, fatigue, disturbed sleep, shortness of breath, coughing, sadness and depression; Physical interference score means average score of sadness and depression; Physical interference score means average score of MDASI-LC interference items of general activity, work, walking; Affective interference score means average score of MDASI-LC interference items of mood, relations with others, and enjoyment of life. QLQ-C30, EORTC Core quality of life questionnaire.

Abbreviations: CI, confidence interval

**Table S3** Clinician Acceptability

| Question | After Trial  Median (Range) Score |
| --- | --- |
| 1. To what extent do you know PRO? | 5(4,5) |
| 2. Do you think the PRO-based symptom management approach is necessary? | 5(1,5) |
| 3. Would you like to recommend this approach (PRO-based) symptom management) to other patients? | 5(5,5) |
| 4. Would you like to recommend this approach (PRO-based) symptom management) to other doctors? | 5(5,5) |
| 5. Do you think the introduction of WeChat applet interactive platform is necessary? | 5(5,5) |
| 6. What is your level of satisfaction with the WeChat applet? | 5(5,5) |
| 7. Do you think using WeChat applet interactive platform adds burden on patients? | 3(0,5) |
| 8. Do you think using WeChat applet interactive platform adds burden on doctors? | 2(1,5) |
| 9. Do you think it is helpful for the psychologist to intervene when the patient's emotional symptoms are severe? | 5(5,5) |

NOTE. N=7, Response rates were 100% for all seven participating doctors and all questions. Higher scores represent better outcomes on 0-5-point scales for questions 1–6,9, and lower scores represent better outcomes on 0-5-point scales for questions 7–8.

Abbreviations: PRO, patient-reported outcome

**Table S4** Patient Satisfaction with the Interventions

| Question |  |
| --- | --- |
| 1. Are you satisfied with the symptom management services provided by your doctor and the services in the WeChat applet? | satisfied 100% |
| 2. Please grade the management services provided by doctors and WeChat applet | Median (Range) Score  5（5,5） |
| 3. Do you feel that filling out the applet questionnaire has disturbed your daily life？ | Completely undisturbed or slightly disturbed 87.41% |
| 4. If you have received a call from an expert, do you think you will feel more relaxed after the chat？ | Received calls: 51.72%  Feel relax 97.27% (completely relaxed: 72.97%%; some relaxation: 24.32%; no change: 2.70% |

NOTE. N=143. Response rates were 143/182=78.57% for all questions. Higher scores represent better outcomes on 0-5-point scales for questions 2. Abbreviations: PRO, patient-reported outcome


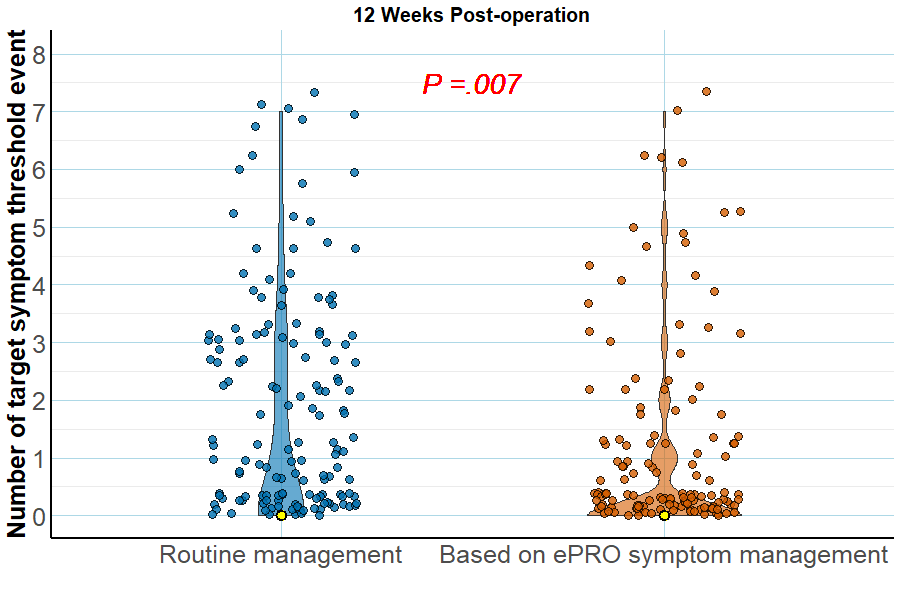


**Figure S1** Intention-to-treat analyses for the number of symptom threshold events 12 weeks post-operation. The violin plot shows the median (Yellow round dot in the box), 25th and 75th quartiles (box limits), PRO, patient reported outcome; IQR, interquartile range.


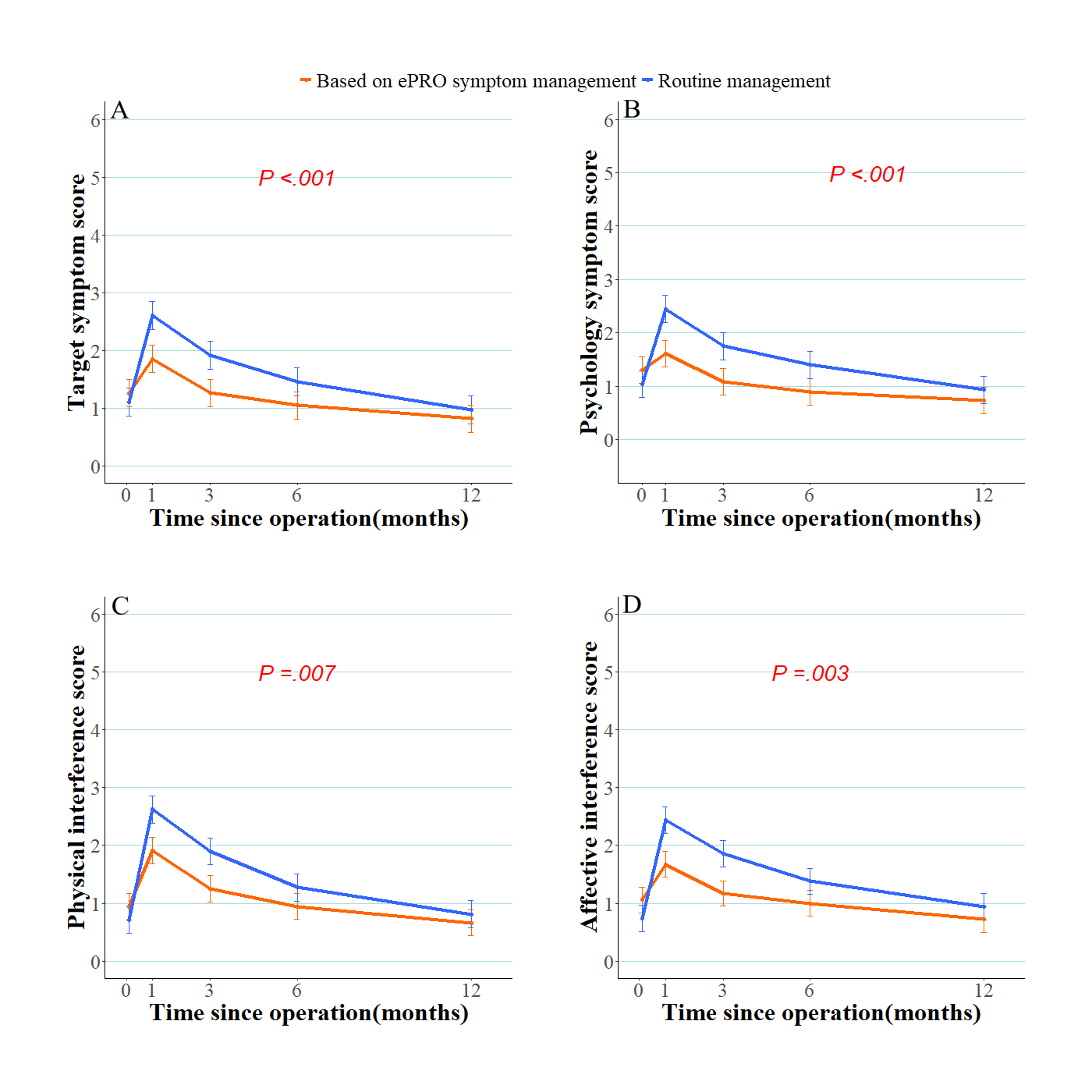


**Figure S2** Symptom severity over time. (A)Target symptom scores. (B)Psychology symptoms score (sadness and distress). (C)Physical interference score (D) Affective interference score. High scores indicate more severe functional interference. I bars represent 95% CIs. MDASI-LC, MD Anderson Symptom Inventory-Lung Cancer module; PRO, patient-reported outcome. 0, baseline, within one week before surgery.


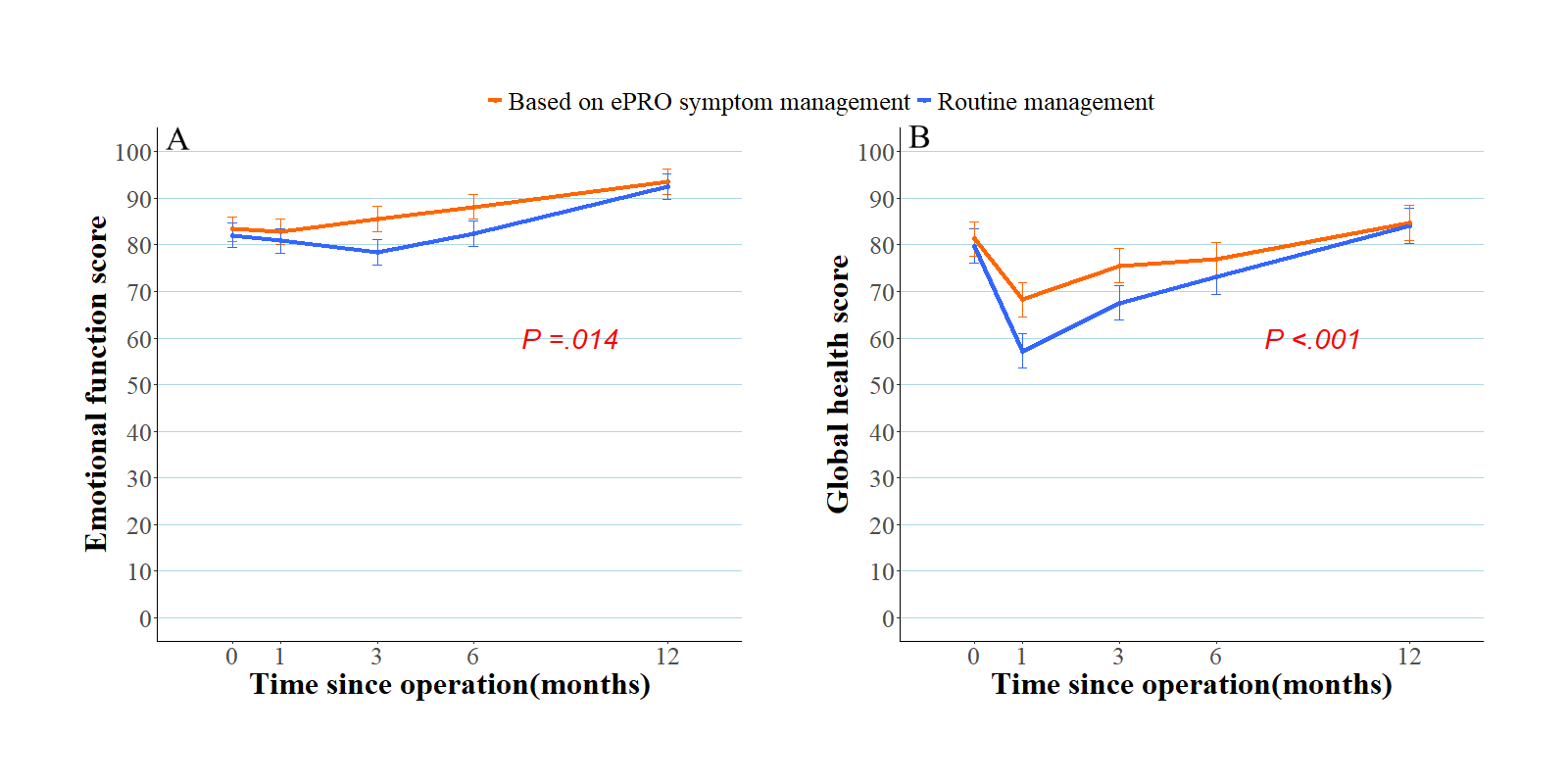


**Figure S3** Emotional function high scores indicate more better emotional functional. Global health high scores indicate better health. I bars represent 95% CIs. QLQ-C30, EORTC Core quality of life questionnaire; PRO, patient-reported outcome; The QLQ-C30 dimension score of Y-axis is standardized score. 0, baseline(within one week before surgery).
